# Supplementary material for: A Fully Human IgE Specific for CD38 as a Potential Therapy for Multiple Myeloma
Source: Cancers (Basel). 2023 Sep 13;15(18):4533. doi: 10.3390/cancers15184533 (PMC10526502; doi:10.3390/cancers15184533)
Supplement: Supplementary file 1 [file cancers-15-04533-s001.zip › cancers-2516361-supplementary.pdf]

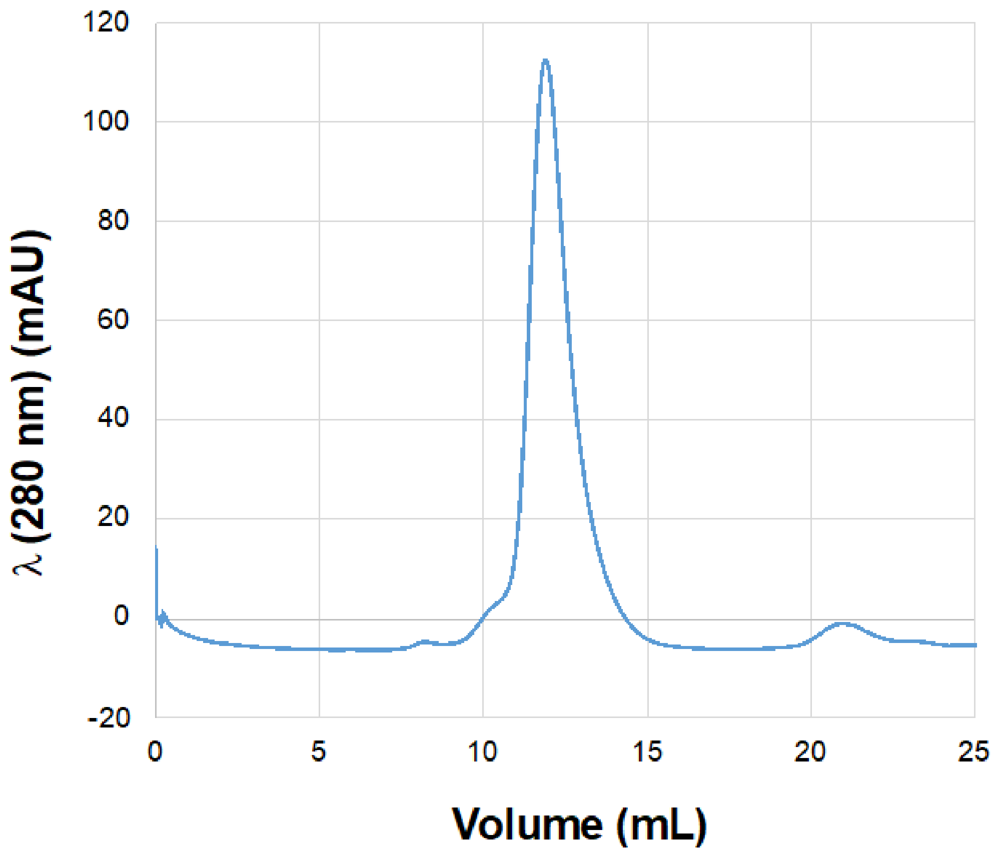

Figure S1. SEC analysis of the anti-CD38 IgE. A 500 μL sample (300 μg) of the antibody was injected on a Superdex 200 10/300 GL size exclusion column equilibrated with protein buffer (150 mM NaCl, 50 mM Tris-HCl; pH 7.8) using a flow rate of 0.5 mL/min on a Bio-Rad NGC chromatography system. This analysis shows that the IgE is monomeric and non-aggregated.

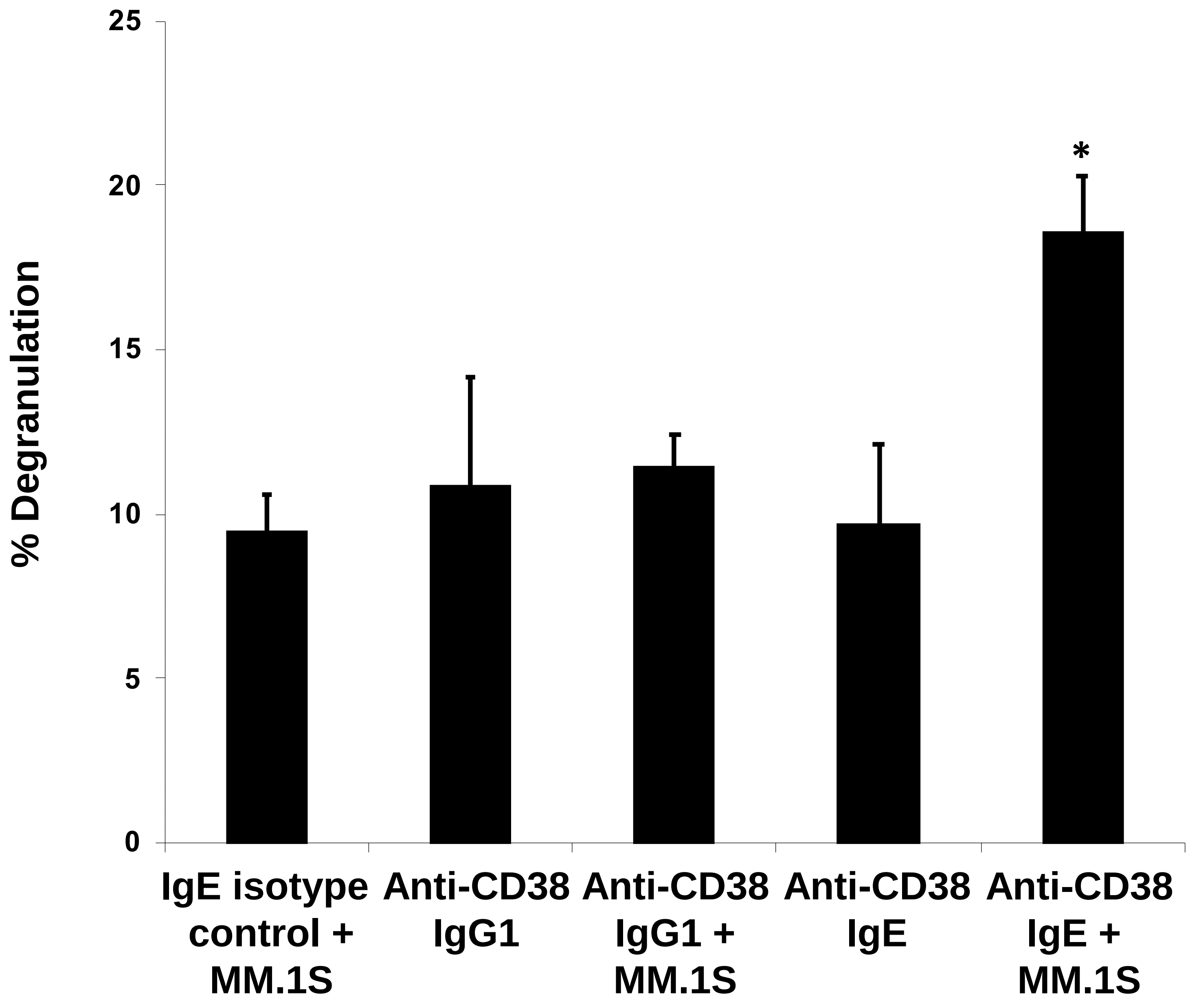

Figure S2. In vitro degranulation assay (replicate study). RBL SX-38 cells were sensitized with 1 μg of either anti-CD38 IgG1, anti-CD38 IgE, or isotype IgE control for 2 h. Supernatant was then replaced with either buffer or MM.1S cells. Release of β-hexosaminidase in the supernatant was measured enzymatically. * p < 0.05 (Student's *t*-test) compared to each control group. The mean and standard deviation of triplicate samples are shown. This is a replicate study of the one shown in Figure 3.

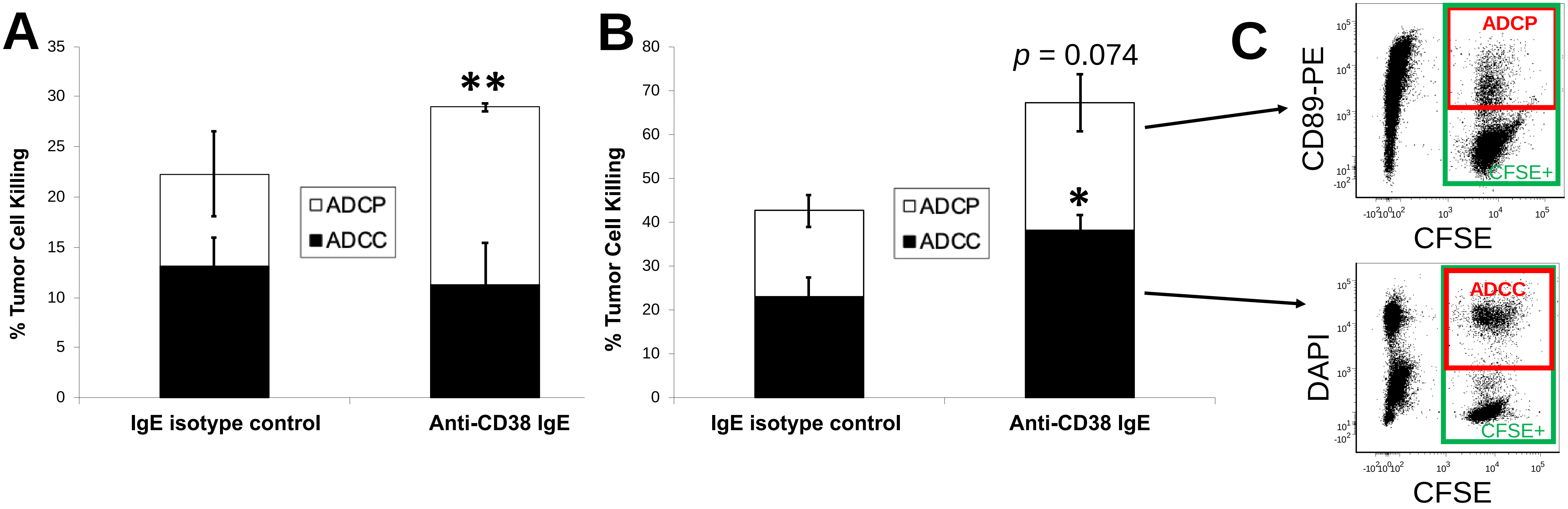

Figure S3. ADCC/ADCP assessed by three-color flow cytometry (replicate studies)**.** Monocytes are isolated and either (**A**) treated with 10 ng/mL of IL-4 for 20 h or (**B**) differentiated into macrophages and activated towards an M1 phenotype, then used as effector cells against CFSE labeled MM.1S target cells treated with either IgE isotype control or anti-CD38 IgE antibody. After incubating effector and target cells at 5:1 effector to target ratio for 2.5 h, cells were stained with both a PE-conjugated mouse anti-human CD89 antibody and DAPI, then analyzed by flow cytometry (5 x 10^4 events were collected). ADCP was defined as CD89-PE$^+$ and CFSE$^+$ events while ADCC was defined as CFSE$^+$ and DAPI$^+$ events. Groups were compared using Student's *t*-test (* $p < 0.05$, ** $p < 0.01$). (**C**) Representative dot plots showing the data presented in Panel B for cells treated with the anti-CD38 IgE.

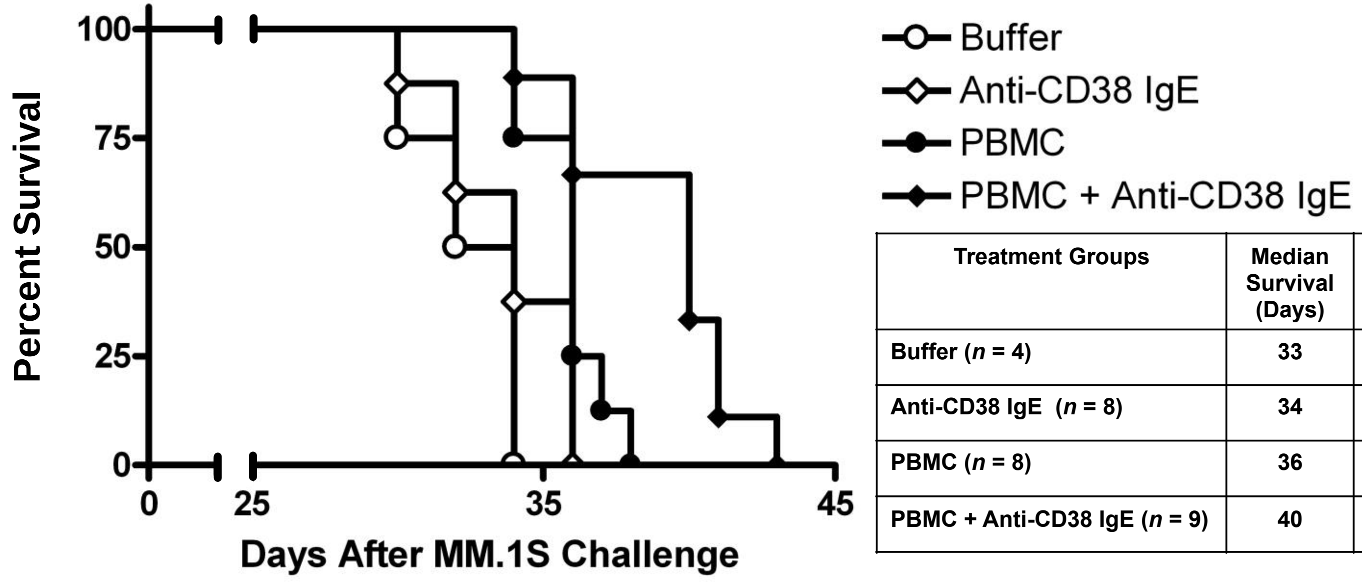

Treatment Groups	Median Survival (Days)	*p*-value vs Buffer	*p*-value vs anti-CD38 IgE	*p*-value vs PBMC
Buffer (*n* = 4)	33	-		
Anti-CD38 IgE (*n* = 8)	34	0.2581		
PBMC (*n* = 8)	36	0.0048*	0.0452*	
PBMC + Anti-CD38 IgE (*n* = 9)	40	0.0009*	0.0021*	0.0080*

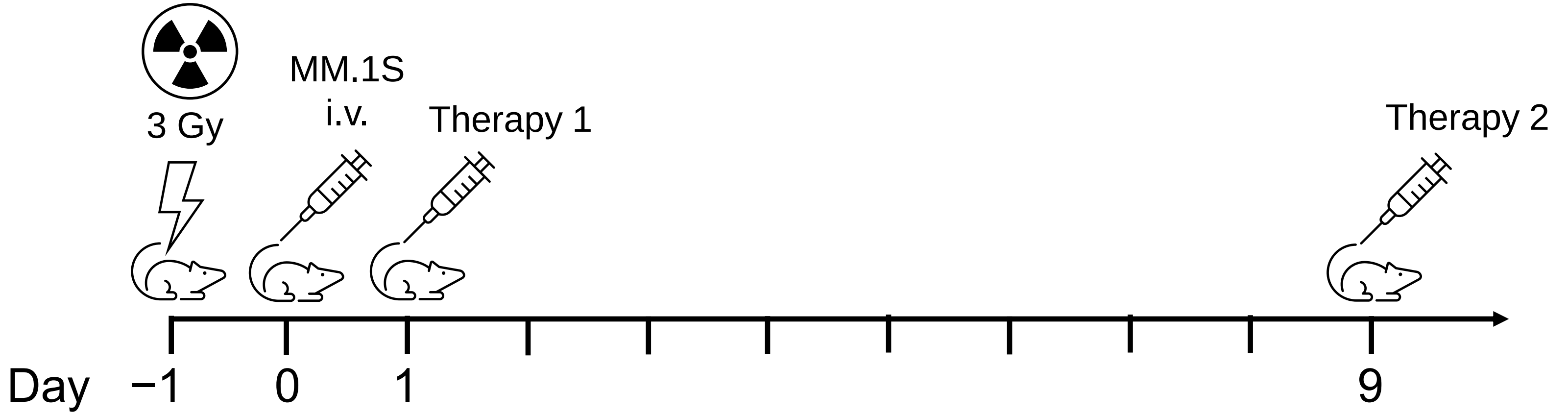

Figure S4. In vivo anti-tumor activity (replicate study)**.** SCID-Beige female mice were whole body irradiated (3 Gray) on Day −1 and on Day 0 were implanted with 5 x 10^6 MM.1S (human MM) cells i.v. On Day 1 and Day 9 post-cell implant mice were treated with buffer control (*n* = 4), 5 x 10^6 PBMC (*n* = 8), 100 µg of anti-CD38 IgE (*n* = 8) or 100 µg of anti-CD38 IgE with 5 x 10^6 PBMC i.v (*n* = 9). Mice were then observed for hind-limb paralysis (end point). Differences between survival curves were calculated using the log-rank test (* $p < 0.05$).
